# Preliminary data for performance in hue ordering tests during pregnancy

**DOI:** 10.1590/1414-431X20187559

**Published:** 2019-01-24

**Authors:** T.S.S. Calandrini, L. Miquilini, M.R. Laranjeiras-Neto, M.T.S. Tongu, M.P. Silva, G.S. Souza, M.I.T. Cortes

**Affiliations:** 1 Programa de Pós-Graduação em Ciências da Saúde, Universidade Federal do Amapá, Macapá, AP, Brasil; 2 Núcleo de Medicina Tropical, Universidade Federal do Pará, Belém, PA, Brasil

**Keywords:** Pregnancy, Visual system, Color vision, Psychophysics

## Abstract

The visual system of women changes during pregnancy. Few reports have addressed the effects of pregnancy on color vision. We aimed to compare the color vision of women in the first, second, and third trimesters of pregnancy. Fifty women were divided into first (n=10), second (n=10), third trimester pregnancy groups (n=10), and non-pregnant group (n=20). We used the Farnsworth D15 and Lanthony desaturated D15 (D15d) tests. The hue ordering quantified the amount of error (C-index) and the chromatic selectivity of the errors (S-index). Bland-Altman analysis was applied to the hue ordering data. No difference was found for Farnsworth D15 test results obtained from the pregnant groups and the non-pregnant group (P<0.0083). For the Lanthony D15 desaturated test, the third trimester pregnant group had higher C-index and S-index than non-pregnant women and first-trimester pregnant women (P<0.0083). The Bland-Altman analysis showed that the limits of agreement increased as pregnancy advanced, and the errors were biased to the D15d test. In this study, color vision was impaired during pregnancy. Color vision evaluation could be used as an indicator of the functional status of the central vision during pregnancy.

## Introduction

Pregnancy is a period during which women experience many changes in their bodies. Endocrine (1), respiratory (2), hematological (3), metabolic (4), visual (5–6), and cardiovascular (7) adaptations alter the structure and function of the whole body to allow the development of the fetus (8). A continuous increase of estrogens, progesterone, and glucocorticoids occurs, which are directly involved with tissue changes (9–10).

Some of these changes, which return to normal after delivery or the breastfeeding period, occur in the different structures and functions of the visual system. During pregnancy, optical and neural modifications seem to alter the visual function. Several studies show an increase in corneal thickness (11) and curvature (12) and a decrease in corneal sensitivity (13). However, some reports found no change in corneal thickness and sensitivity (14). Intraocular pressure decreases mainly in the last trimester of pregnancy (11,15–16). Visual accommodation loss is observed during pregnancy and breastfeeding (17). All these optical changes can lead to visual acuity impairment and visual field constriction (18). Neural changes have been observed in visual evoked potential studies. Pregnant women had shorter latency responses than non-pregnant women (19). The physiological consequences of these faster responses in pregnant women are not clear.

Also, a previously published study investigated the influence of pregnancy on color vision. It was observed that first-trimester pregnant women had better performance in the hue ordering test than non-pregnant women (20). The authors explained that the difference was due to the hormonal status of the pregnant women that would be similar to the status of non-pregnant women in the ovulatory stage of the menstrual cycle (21). However, only first-trimester pregnant women were investigated. As many effects of pregnancy are cumulative as time progresses, it would be interesting to evaluate if during the other trimesters these effects would have the same influence on color vision.

Therefore, we investigated color vision changes in the three trimesters of pregnancy. We also applied two different tests to evaluate the hue ordering performance: Farnsworth D15 test to investigate severe color vision loss, and Lanthony desaturated D15 (D15d) test to investigate mild color vision loss.

## Material and Methods

### Subjects

The study was carried out with thirty pregnant women (26.3±7.4 years old) and twenty non-pregnant women (27.65±6.54 years old). The pregnancy was confirmed by a blood test, and the period of pregnancy was determined by ultrasound imaging and self-report about the last menstrual period. All participants were recruited in the Basic Health Unit of the Federal University of Amapá, where they were receiving antenatal assistance.

The pregnant women were divided into three groups: *i*) first-trimester group included women with pregnancy duration between 1 and 13 weeks (n=10; 11.3±1.95 weeks); *ii*) second-trimester group included women who were 14–26-week pregnant (n=10; 19.7±3.05 weeks); and *iii*) third-trimester group included women with a pregnancy duration from 27–40 weeks (n=10; 31.2±2.97 weeks). In the control group, women with 23–35-day menstrual cycle (22), in any stage of the cycle were included; there was no restriction about the use of hormonal contraceptives. In the control, 12 women used non-hormonal contraceptive methods, five used oral contraceptive methods such as levonorgestrel, ethinylestradiol, and cyproterone acetate, and three used injected contraceptive methods such as norethisterone enanthate, estradiol valerate, algestone acetophenide, and estradiol enanthate.

All patients underwent ophthalmologic examinations, including visual acuity, refractometry, fundoscopy, and tonometry. The red-green color vision phenotype was determined by the Ishihara test.

We excluded participants with a Snellen visual acuity higher than 20/40, more than 8 errors in the Ishihara test, previous chronic exposure to organic solvents or toxic heavy metals, and ophthalmic, neurological or systemic diseases that may affect the visual system. None of the pregnant women had developed diabetes or hypertension before or during pregnancy and none were at risk.

All procedures had the approval of the Research and Ethics Committee of the Federal University of Amapá (report #52734115.0.0000.003), and informed consent from each patient was obtained.

### Color vision tests

Color vision was evaluated, using the Farnsworth D15 test (Richmond Products, USA) and the D15d test (Richmond Products). Both color arrangement tests were composed of 16 caps (1 reference cap and 15 test caps). The difference between both tests is that Farnsworth D15 has caps with more saturated chromaticities than D15d. These caps were shown at 60 cm under a daylight illuminator for color vision testing (5733R model, 6280 K daylight, Richmond Products). The ambient illuminance level was 300 lux measured using a PR-650 SpectraScan colorimeter (Photo Research Inc., USA) at 70 cm of the illuminator.

For each test, the correct arrangement of the caps was shown to the observer for 1 min. Then, we shuffled the caps without the observation of the participant. The participants were instructed to organize the caps following the similarity between the hues of neighbor caps starting by the reference cap, such as the caps arrangement previously shown. The task was to arrange the caps in the correct order, according to hue clues from the reference cap. The participant was asked about her comprehension about the task to be done. After completing the task, we asked if the arrangement was correct or if any change in cap arrangement was still needed. The test was finished after the participant confirmed that the caps arrangement was correct for her. One trial for each eye was conducted. The sequence of arrangement was analyzed by the Vingrys and King-Smith method (23) with the C-index and the S-index as visual outcomes. The test duration was about 7 min.

### Data analysis

For the data analysis, we chose the outcomes (C-index, S-index, and angle) obtained from the eye with the lower C-index (best hue ordering performance) for each test. Statistical analysis was performed using the software Biostat v 5.3 (Brazil). For each parameter, we tested the normality of the data using the Shapiro-Wilk test. For the intergroup comparison, we used the Mann-Whitney test followed by the Bonferroni correction. We considered the level of significance corrected to multiple comparisons at 0.083%. The effect size was calculated using Cohen's d calculator.

We also used Bland-Altman plots to evaluate the agreement of the D15d test and Farnsworth D15 test for all the groups.

## Results

Table 1 shows the descriptive statistics of the outcomes estimated from the hue ordering tests. For D15d test, the multiple comparisons showed that the third-trimester group had more error (higher C-Index) than the controls (one-tailed Mann-Whitney test, P<0.0083). We chose the one-tailed analysis because the perfect result that is expected for normal trichromats (control) is 1 and worsening of the performance would only increase the outcome. The effect size measured by Cohen's d for this difference was 1.286. No other multiple comparison of C-index values had significant difference (P>0.0083). We also observed that the S-index of the third-trimester group was greater than controls (two-tailed Mann-Whitney test, P<0.0083). In the case of the S-index, we considered the two-tailed analysis, because the S-index of the test groups can be higher or lower than the controls. The effect size measured by Cohen's d for this difference was 1.835. There was no difference between the angle of the error estimated from the controls and the third-trimester group. No other multiple comparisons of S-index and angle showed significant differences. For Farnsworth D15 test, the multiple comparisons showed no difference among C-index, S-index, or angle of error estimated from the groups.

**Table 1.** Descriptive statistics of the hue ordering test results of first to third-semester pregnant women and controls.

	Lanthony D15d	Farnsworth D15
C-index		
Control	1 (1–1.08)	1 (1–1)
1st trimester	1 (1–1)	1 (1–1)
2nd trimester	1.10 (1.02–1.19)	1 (1–1)
3rd trimester	1.27 (1.08–1.66)*	1.03 (1–1.2)
S-index		
Control	1.49 (1.49–1.49)	1.49 (1.49–1.49)
1st trimester	1.49 (1.49–1.49)	1.49 (1.49–1.49)
2nd trimester	1.49 (1.49–1.59)	1.49 (1.49–1.49)
3rd trimester	1.74 (1.61–2.08)*	1.49 (1.49–1.77)
Angle (degree)		
Control	61.56 (61.56–61.56)	61.56 (61.56–61.56)
1st trimester	61.56 (61.56–61.56)	61.56 (61.56–61.56)
2nd trimester	66.20 (61.79–71.73)	61.56 (61.56–61.56)
3rd trimester	61.56 (60.86–71.35)	61.56 (61.57–65.50)

The data are reported as median (first quartile – third quartile). *P<0.083 compared to Control (one-tailed Mann-Whitney test).

**Figure 1.**
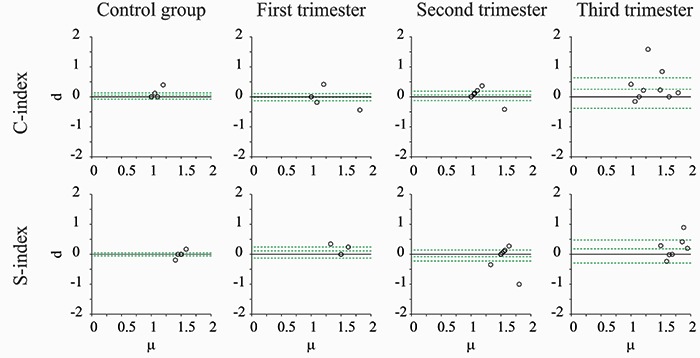
Bland-Altman plots for C-index (upper panels) and S-index (lower panels) to evaluate the agreement between Lanthony D15d and Farnsworth D15 tests. The Y-axis represents the difference between the measurements obtained from both tests (d) and the X-axis represents the mean value between the measurements obtained from both methods (μ). Plots for the control, first trimester, second trimester, and third trimester groups are shown from left to right, respectively. Circles represent the database, upper green dotted line represents the upper limit of agreement, lower green dotted line represents the lower limit of agreement, and the intermediate green dotted line represent the mean difference observed between both methods. The black solid line represents the zero difference.

Figure 1 shows the Bland-Altman plots for C-index and S-index values. For both indexes, the confidence interval of the difference between both measurements became greater with pregnancy progression. Especially at the third trimester of pregnancy, the measurements were biased to the D15d.

## Discussion

The present investigation showed that color vision was impaired during pregnancy. At the beginning of pregnancy, the color vision is similar to the non-pregnant group color vision, but with pregnancy progression, the number of errors (C-index) in the color arrangement test became higher, reaching the worst performance in the third-trimester.

The color vision loss had no color preference at any stage of pregnancy because the angle of error during the different stages of the pregnancy was similar to that obtained in control women. This seems expected because the existence of some adverse condition during the pregnancy must be non-selective for the different chromatic mechanisms.

A previous investigation compared the color vision of first-trimester pregnant to non-pregnant women (20). The investigators used the Farnsworth-Munsell 100 hue test to evaluate the color vision of the sample. They found that first-trimester pregnant women had better performance than control women. The authors interpreted that during pregnancy, the maintenance of high levels of estrogen could increase the color vision performance, similar to that observed during the ovulatory phase of the menstrual cycle. We found a similarity of color vision performance between non-pregnant women and pregnant women using Farnsworth D15 test, a short version of the Farnsworth-Munsell 100 hue test. We have no clear explanation for the differences between our and Orbán and Dastur's investigation (20). The main differences were that the change occurs more slightly in the Farnsworth-Munsell 100 hue test than in Farnsworth D15 test, and thus, the latter could enable to find small differences between the first-trimester pregnant and non-pregnant women. Both studies had similar sample sizes and age range of the control and first-trimester pregnant women. The control groups were different, because we included women using contraceptive hormonal therapy, while Orbán and Dastur (20) had only women without contraceptive therapy. We considered that there was no evidence of negative influence from the contraceptive hormone therapy in the color vision (24) and all participants had normal results in the ophthalmological examination. The functional alteration was found only in the desaturated test, Lanthony D15d test, indicating a mild color vision loss. The possible protective effect of estrogens during pregnancy cannot explain the color vision deficits we observed in the third-trimester pregnant women. The Bland-Altman analysis showed that the agreement between the measurements estimated from both tests became lower with the advancement of pregnancy and that the errors became biased to the Lanthony D15d test. This result suggested that pregnancy had a mild influence in color vision that impaired the hue ordering of desaturated color, but it had no significant influence in the hue ordering performance of saturated colors.

Our results can be explained by the hormonal status variation (especially those estrogen-related) that occurs during pregnancy and its multiple tissue alterations in the eye. Three physiological estrogens seem to play different roles in the health and disease processes in humans (25). E2, or estradiol, is the predominant physiological estrogen in women before menopause; E1, or estrone, is an estrogen that increases in women after menopause; and E3 is an estrogen present in high concentrations during pregnancy parallel with E2. There is a large amount of estrogen receptor genes, of which the expression in the eye is influenced by gender and age, but the mechanisms in which they are involved, are still unclear (26). Evidence shows the protective effect of estrogen in women's vision. Postmenopausal women have a greater incidence of maculopathy and cataracts (27), which is reduced after estrogen replacement therapy (28). To explain our results, we considered two possible mechanisms, resulting from the pregnancy hormonal status: changes in the eye optics and changes in the choroidal function.

The increased corneal thickness and water retention in the corneal tissues are associated with the increased curvature of the lens, resulting in a higher refractive error that leads to transient myopia during pregnancy (29). The influence of myopia in color vision is usually weak, but it could be similar to what has been observed in highly myopic subjects without retinal degeneration (30). Myopic subjects performed several color vision tests normally, but for box III of Farnsworth-Munsell 100 hue test (bluish caps), they had a higher error of color arrangement than normal subjects.

The subfoveal choroidal thickness was observed to be increased during pregnancy, probably because of the increase in the blood flow in the choroidal vessels (31). Choroidal vasodilation leads to a vascular hyper-permeability followed by vascular leakage into the retinal tissue, which could impair the metabolizing exchanges in the avascular fovea (32). As the fovea is the main region of the retina that underlies the human color vision, any condition that generates an increase in the metabolic stress could impair this visual function.

Previous studies and the present investigation used hue ordering tests to evaluate the color vision of pregnant women. Hue ordering is a color vision-dependent task that estimates supra-threshold performance of the visual system. Other tests, such as Cambridge Colour Test or Colour Assessement Diagnosis, that evaluate color discrimination threshold, are candidates for future investigations of pregnancy effects in color vision (33). There is a debate about the reliability of the Lanthony D15-d when used only once, because a considerable within-subject variability was observed (34). Our participants carried out one trial after a thorough explanation about the test, and after completing the task, subjects were asked if they would like to change the position of any cap. As all the comparisons were done in the same conditions, the differences found should be due to different features of the groups.

Our control group was composed by non-pregnant women in different stages of the menstrual cycle and that used different contraceptive methods. The literature findings are controversial about the visual functions during the different stages of the menstrual cycle or between women that used or not contraceptive methods (21,35). We considered that the composition of our groups was a limitation of the study, but with no or few impacts in the results. Pregnancy is characterized by the presence of changes in emotional regulation (36), cognitive functioning (37), and comorbidities (38), which can affect visual processing. We controlled the presence of comorbidities. Davies et al. (39) undertook a meta-analysis about the quantitative relationship between pregnancy and changes in cognition and executive functions. They found that pregnant women had impairment of cognitive function, particularly during the third trimester of pregnancy. Moreover, they asserted that the results should be interpreted with caution because the cognitive decline is significant, but remains in a normal range. Considering our results, we did not exclude an influence of cognitive and motivational decline in the performance of pregnant women in the color vision tests, but it was not clear how strong the influence was. As the test we used is ludic, quick, and easily understandable, we suggest that the global influence of cognitive impairments on the hue ordering test should be small.

We did not analyze emotional regulation, cognitive functioning, memory, attention, or psychomotor speed. None of the participants had a clinical complaint during the period of tests.

The present investigation had some limitations. We considered that a longitudinal research design, following the same patients in the first, second, and third trimesters, would make our conclusions stronger.

Visual complaints are common in pregnant women, and they and their physicians should pay attention to those. Considering that most visual complications of pregnancy are harmless, have transitory time-course and no treatment is needed, our results are relevant for raising the awareness of the existence of color perception impairment even in healthy pregnancies, and to help differ from acquired color vision deficiencies that may arise from systemic diseases such as systemic arterial hypertension or diabetes during the pregnancy. The clinical relevance of our results was that the color vision of pregnant women can be used as an important indicator about the nervous system status during pregnancy. It is not clear if the mild color vision impairment we found would affect pregnancy behavior, since third-trimester pregnant women naturally decrease their occupational activities and the deficiency can be bypassed during this period. These tests could be carried out as part of antenatal care and after delivery in order to check the recovery of the visual system to baseline levels.

Color vision was impaired during pregnancy, as a consequence of changes in the eye optics and in the subfoveal choroidal thickness. Due to the mildness of the deficiency, it is rarely a concern for pregnant women and antenatal care. Color vision level could be used as indicator of the central vision functional status during pregnancy.
